# Sociodemographic, clinical, and genetic factors associated with self-reported antidepressant response outcomes in the UK Biobank

**DOI:** 10.1017/S0033291725000388

**Published:** 2025-03-12

**Authors:** Michelle Kamp, Chris Wai Hang Lo, Grigorios Kokkinidis, Mimansa Chauhan, Alexandra C. Gillett, AMBER Research Team, Andrew M. McIntosh, Oliver Pain, Cathryn M. Lewis

**Affiliations:** 1 Social, Genetic and Developmental Psychiatry Centre, Institute of Psychiatry, Psychology & Neuroscience, King’s College London, London, UK; 2 NIHR Maudsley Biomedical Research Centre, South London and Maudsley NHS Trust, London, UK; 3 Division of Psychiatry, University of Edinburgh, Edinburgh, UK; 4 Department of Basic and Clinical Neuroscience, Institute of Psychiatry, Psychology and Neuroscience, King’s College London, London, UK; 5 Department of Medical and Molecular Genetics, Faculty of Life Sciences and Medicine, King’s College London, London, UK

**Keywords:** antidepressant response, CYP2C19 metabolizer status, major depressive disorder (MDD), polygenic scores, selective serotonin reuptake inhibitors (SSRIs)

## Abstract

**Background:**

In major depressive disorder (MDD), only ~35% achieve remission after first-line antidepressant therapy. Using UK Biobank data, we identify sociodemographic, clinical, and genetic predictors of antidepressant response through self-reported outcomes, aiming to inform personalized treatment strategies.

**Methods:**

In UK Biobank Mental Health Questionnaire 2, participants with MDD reported whether specific antidepressants helped them. We tested whether retrospective lifetime response to four selective serotonin reuptake inhibitors (SSRIs) (*N* = 19,516) – citalopram (*N* = 8335), fluoxetine (*N* = 8476), paroxetine (*N* = 2297) and sertraline (*N* = 5883) – was associated with sociodemographic (e.g. age, gender) and clinical factors (e.g. episode duration). Genetic analyses evaluated the association between CYP2C19 variation and self-reported response, while polygenic score (PGS) analysis assessed whether genetic predisposition to psychiatric disorders and antidepressant response predicted self-reported SSRI outcomes.

**Results:**

71%–77% of participants reported positive responses to SSRIs. Non-response was significantly associated with alcohol and illicit drug use (OR = 1.59, *p* = 2.23 × 10^−20^), male gender (OR = 1.25, *p* = 8.29 × 10^−08^), and lower-income (OR = 1.35, *p* = 4.22 × 10^−07^). The worst episode lasting over 2 years (OR = 1.93, *p* = 3.87 × 10^−16^) and no mood improvement from positive events (OR = 1.35, *p* = 2.37 × 10^−07^) were also associated with non-response. CYP2C19 poor metabolizers had nominally higher non-response rates (OR = 1.31, *p* = 1.77 × 10^−02^). Higher PGS for depression (OR = 1.08, *p* = 3.37 × 10^−05^) predicted negative SSRI outcomes after multiple testing corrections.

**Conclusions:**

Self-reported antidepressant response in the UK Biobank is influenced by sociodemographic, clinical, and genetic factors, mirroring clinical response measures. While positive outcomes are more frequent than remission reported in clinical trials, these self-reports replicate known treatment associations, suggesting they capture meaningful aspects of antidepressant effectiveness from the patient’s perspective.

## Introduction

Major depressive disorder (MDD) is a prevalent and debilitating condition characterized by persistent low mood, loss of interest, cognitive impairment, and physical symptoms such as disrupted sleep or appetite (Otte et al., 2016). Affecting approximately one in six adults globally, MDD incidence continues to rise annually (Abdoli et al., 2022; GBD 2019). In 2018, the World Health Organization (WHO) ranked MDD third in global disease burden and predicted it will be the leading cause by 2030 (Cui et al., 2024).

Antidepressants, specifically selective serotonin reuptake inhibitors (SSRIs), are first-line pharmacological treatments for MDD (Bauer, Severus, Möller, & Young, 2017; Cleare et al., 2015; NICE, 2009). In England’s National Health Service (NHS), antidepressant prescriptions nearly doubled from 36 million in 2008 to 70.9 million in 2018 (Iacobucci, 2019). SSRIs have similar or greater efficacy than other antidepressants and are preferred clinically for their fewer side effects (Cipriani et al., 2018; Karrouri, Hammani, Benjelloun, & Otheman, 2021). However, antidepressant efficacy varies with only ~35% of patients achieving remission after initial treatment (Rush et al., 2006), and approximately 40% developing treatment-resistant depression, defined as the lack of response to two or more antidepressants in the same depressive episode (Rush et al., 2011; Souery et al., 2011).

Variability in treatment response may be due to disorder heterogeneity (Cui et al., 2024; Fried & Nesse, 2015), genetics (Pain et al., 2022; Tansey et al., 2013), and sociodemographic or clinical factors (Perna et al., 2020). While studies assessing the impact of age and sex on antidepressant response show inconsistent results (Kessler et al., 2017; Khan et al., 2005; Perna et al., 2020; Saveanu et al., 2015; Trivedi et al., 2006); socioeconomic factors such as low income and unemployment have been associated with poor response to the antidepressant citalopram (Trivedi et al., 2006). Genome-wide association studies (GWASs) have yet to find robust genetic predictors of antidepressant response, likely due to small sample sizes, study design, and drug and outcome heterogeneity (Ising et al., 2009; Ji et al., 2013; Uher et al., 2009). Nevertheless, SNP-based heritability estimates by the Psychiatric Genomics Consortium suggest that 13% to 40% of the variance in antidepressant response is attributable to common genetic variation (Pain et al., 2022).

Pharmacogenetic studies have identified variations in the cytochrome P450 (CYP) enzyme superfamily that affects drug response via pharmacokinetic mechanisms. Polymorphisms in *CYP* genes, including *CYP2C19*, impact enzyme activity and may explain individual differences in treatment response (Li et al., 2024; Wong et al., 2023). However, CYP2C19 and other candidate genes account for only a small fraction of variability in drug response. Polygenic scores (PGS) offer an alternative by quantifying an individual’s genetic predisposition to a trait or disease, aggregating the effects of multiple SNPs identified through GWAS. By capturing the polygenic nature of treatment response, where many loci contribute small effects, PGS may be valuable for predicting response. Although PGS for bipolar disorder and MDD, based on relatively small sample sizes, have shown inconsistent associations with treatment outcomes (Fanelli et al., 2021; García-González et al., 2017), positive antidepressant response has been associated with low genetic liability for schizophrenia (Pain et al., 2022).

The trial-and-error approach to finding the right antidepressant delays recovery, increases side effects, reduces adherence, and highlights the need to identify individual moderators of treatment response to support personalized treatments (Perna et al., 2020). Most studies identifying factors associated with antidepressant response come from clinical trials, which often have limited generalizability due to restrictive inclusion criteria and controlled settings. However, comprehensive datasets, including retrospective self-reports on antidepressant response, are becoming available (Koch et al., 2024). Little is known about predictors associated with an SSRI-user reporting that an antidepressant ‘helped’ them. This study uses retrospective self-report data from approximately 20,000 UK Biobank participants to assess sociodemographic, clinical, and genetic predictors of this patient-focused measure of SSRI response and compare them with those identified in prospective clinical studies.

## Method

### Participants

The UK Biobank (UKB) is a large-scale research resource investigating the impact of genetic, environmental, and lifestyle factors on health outcomes in middle-aged and older adults. Individuals aged 40–69 registered with the UK National Health Service were invited to participate, and 500,000 individuals were recruited in 22 assessment centers across the UK between 2006 and 2010 (Allen, Sudlow, Peakman, & Collins, 2014). Baseline data included sociodemographic characteristics, medical histories, and health and lifestyle factors. Two online mental health questionnaires have been issued. The second questionnaire (MHQ2; Category 1502), which was initiated in 2022 and completed by 172,912 participants, included information on treatment (MHQ2 resources are available at https://osf.io/c65t7/). Participants who endorsed at least one of two cardinal lifetime MDD symptom screening questions (UKB fields 29011 and 29012, Supplementary Data S1, Supplementary Table S1) completed the medication and antidepressant response sections (*N* = 79,888). After excluding participants with prior or probable schizophrenia, bipolar, or mania diagnoses (*N* = 1527, from UKB fields 29000 and 20126) and those missing genetic information (1835 individuals), 76,526 individuals with unipolar MDD were available for this analysis.

Prescription medication users (Yes/No) (*N* = 35,088) were identified as those who reported trying prescribed medication for low mood or anhedonia (UKB field 29038, Supplementary Data S1, Supplementary Table S1). Antidepressant users were prescription medication users who reported trying specific antidepressants for at least 2 weeks (UKB field 29039), with four SSRIs listed: citalopram, fluoxetine, paroxetine, or sertraline (*N* = 20,613) (Supplementary Data S1, Supplementary Figure S1). SSRIs were limited to these four drugs as they were the options listed in MHQ2, aligning with prescription records showing they account for over 90% of prescribed antidepressants in the UK: citalopram (36%), fluoxetine (33%), paroxetine (12%), and sertraline (15%) (Lo et al., 2024).

### Ethics and consent

The UK Biobank has research ethics approval from the North West Multi-center Research Ethics Committee (MREC; approval number 11/NW/0382) covering the UK. Participation is voluntary, and participants can withdraw at any time. Informed written consent was obtained at baseline. The current study was performed under UK Biobank application 82087. All relevant ethical guidelines have been followed during the analysis.

### Measures

#### Self-reported antidepressant response outcomes

The antidepressant response was restricted to 19,516 SSRI users who reported “Yes, even a little (Y)” or “No (N)” to whether at least one of the SSRIs – citalopram, fluoxetine, paroxetine, sertraline – had helped them “feel better” (UKB field 29040, 29041, 29042, and 29043). Those who responded “Do not know” and “Prefer not to answer” were excluded.

##### SSRI antidepressant response

The primary outcome for this study was SSRI response, coded as Y/N. For participants using multiple SSRIs, consistent responses across antidepressants (i.e. all “Y,” or all “N”) were categorized “Y” or “N” accordingly. For any participant reporting “N” for any antidepressant their composite-SSRI response was “N.” In all analyses, the reference category for SSRI response was a positive response.

A sensitivity analysis using a binary SSRI-conservative outcome was conducted for participants using multiple SSRIs, assigning a missing value where only one “N” was reported (Supplementary Data S1, Supplementary Table S2). In addition, four binary drug-specific response outcomes were defined as part of a sensitivity analysis for sociodemographic and clinical variables. A positive response was defined as “Y” to where users had responded “Yes, even a little,” to whether the specific SSRI (citalopram, fluoxetine, paroxetine, or sertraline) helped them “feel better.” Each drug-specific response was analyzed independently of other self-reported antidepressant exposures. For genetic variables, namely CYP2C19 metabolizer status and psychiatric disorder PGS (described in *Genetic factors*), drug-specific effects are important and tested as a primary outcome.

#### Sociodemographic factors

Sociodemographic variables collected at baseline UKB assessment were analyzed, including age, sex, ethnicity, educational attainment, household income, and neighborhood deprivation. Age at recruitment was recorded in years. Gender was self-reported as biological sex (female/male). Ethnic background was self-reported as “Asian or Asian British,” “Black or Black British,” “Mixed background,” “White” and “Other ethnic group”; for analysis, categories were grouped as “Asian,” “Black,” “Mixed,” and “White,” with White as the reference.

Educational attainment was classified into five categories based on highest education level achieved (Rayner et al., 2021): ‘Secondary’ for completion of compulsory secondary education (GCSE level, 11 years of education); ‘Further’ for completion of further education (A-levels, 13 years); ‘Vocational’ for a range of vocational and professional qualifications (NVQs, BTECs, Apprenticeships, ≥12 years); and ‘University’ for university-level education (Degree, ≥16 years); ‘None’ for none of those listed as the modal category, University was set as the reference. Annual household income was divided into five categories: Less than £18,000, £18,000–£31,000, £31,000–£52,000, £52,000–£100,000, and Greater than £100,000, with the median category (£30,000–£52,000) serving as the reference.

Neighborhood deprivation was measured using the Townsend Deprivation Index (TDI), which combines four census variables (unemployment, car ownership, homeownership, household overcrowding), standardized and summed to a total score. Areas with TDI > 0 are more deprived, while TDI < 0 indicates more affluent areas. The variable “illicit drug and alcohol use” was derived from participants’ reports of using drugs and alcohol to manage their two cardinal MDD symptoms as these coping mechanisms have been linked to treatment-seeking behavior for MDD (Rayner et al., 2021).

#### Clinical factors

MDD symptoms during a participant’s worst episode of MDD are available for UKB participants who answered relevant questions derived from the Composite International Diagnostic Interview (CIDI) conducted through the MHQ2, data category 1502 (World Health Organization, 1993). The questions identify key depression symptoms, including persistent sadness, loss of interest or pleasure, changes in weight or sleep, fatigue, guilt, worthlessness, concentration difficulties, and suicidal ideation (World Health Organization, 1993).

The MHQ2 also assessed clinical characteristics of MDD through self-report, including the lifetime number of depressive periods, age at first and last episode, and whether episodes were related to childbirth or trauma. Family history of severe depression was determined if the participant reported their father, mother or sibling experienced severe depression (UKB field 20107, 20110, 20111); as the modal category, “No” was the reference category. The full list of clinical variables, their data fields, and reference categories used in this analysis are in Supplementary Data S1 (Supplementary Table S3).

#### Genetic factors

Genome-wide genotyping is available for all UKB participants. Samples underwent standard quality control (QC) and imputation. Further description of UKB samples, as well as details on genotyping QC and imputation, is available in Supplementary Data S1.

##### CYP2C19 metabolizer status

CYP2C19 is a key enzyme in SSRI metabolism, with genetic variation in the CYP2C19 gene associated with differential metabolic activity and, consequently, differential SSRI exposure. Metabolic capacity is determined by specific allelic variants, with the patient classified into one of five metabolizer groups based on genotypes and corresponding enzymatic function - poor (no functional enzyme), intermediate (reduced enzyme activity), normal (normal enzyme activity), rapid, and ultra-rapid (increased enzyme activity). CYP2C19 genotypes and metabolizer status were obtained from UKB return 3388 as described by McInnes et al. (McInnes et al., 2021). In brief, pharmacogenetic star alleles and metabolizer phenotypes were identified using the Python program PGxPOP, which determines and reports pharmacogenetic star alleles (popular nomenclature that corresponds to functional haplotype patterns that influence drug metabolism) and haplotypes from phased multisample VCFs using PharmCAT allele definition files (https://github.com/PharmGKB/PharmCAT) (Sangkuhl et al., 2020). PGxPOP applies guidelines from the Dutch Pharmacogenetics Working Group (DPWG) and the Clinical Pharmacogenetics Implementation Consortium (CPIC) to correlate haplotypes with predicted metabolic phenotypes. Participants were categorized into five metabolic phenotypes – poor (PM), intermediate (IM), normal (NM), rapid (RM), and ultrarapid (UM) – based on predicted CYP2C19 activity. Individuals with “indeterminate” or “not available” phenotypes were excluded due to unknown, uncertain, or unaligned star allele functions, preventing phenotype assignment.

##### Polygenic scores

Polygenic scores were calculated for five psychiatric conditions and two antidepressant response outcomes using GWAS summary statistics from the Psychiatric Genomics Consortium (Demontis et al., 2019; Grove et al., 2019; Pain et al., 2022; Pardiñas et al., 2018; Stahl et al., 2019; Wray et al., 2018) (Supplementary Data S1, Supplementary Table S4). The psychiatric conditions included depression (DEPR), autism (AUTI), Attention-Deficit/Hyperactivity Disorder (ADHD), bipolar disorder (BIPO), and schizophrenia (SCHI). The two antidepressant response outcomes were percentage improvement (ADperc), and non-remission (ADnon-rem) (Pain et al., 2022). ADperc was calculated as 100*(baseline depression score − final depression score)/baseline depression score, with higher ADperc indicating better treatment response. Remission is a binary measure attained when a patient’s depression symptom score decreases to a pre-specified threshold for the relevant rating scale. Patients who did not reach these thresholds were classified as non-remitting (Pain et al., 2022).

PGS was calculated using the MegaPRS method within the GenoPred Pipeline (https://opain.github.io/GenoPred/) (Pain, Al-Chalabi, & Lewis, 2024). The MegaPRS model selected by its pseudo-summary approach was used in downstream analyses. The pseudo-summary approach estimates the best tuning parameters based solely on the GWAS summary statistics, avoiding the need for an external validation sample. MegaPRS was used due to its superior prediction performance for psychiatric disorders (Ni et al., 2021). Covariates included the first six principal components (to adjust for population stratification), age, sex, and genotyping batch. The assessment center was excluded as a covariate as association testing showed no significant association with self-reported response outcomes (Supplementary Data S1, Supplementary Table S5).

### Statistical analyses

The primary outcome analyzed was self-reported binary SSRI response (Y/N). Separate association analyses assessed associations between sociodemographic, clinical, and genetic predictors with SSRI response using univariable and multivariable logistic regression models and the odds ratio (OR) reported. The reference level was a positive response; hence, all ORs describe the odds of SSRI non-response compared to a positive response. Clinical variables reaching nominal significance were taken forward into multivariable models. For all analyses, variance inflation factors assessed multicollinearity in multivariable models (Supplementary Data S1, Supplementary Tables S9 and S10) and covariates included age and sex. Sensitivity analyses for drug-specific responses are presented in the Data S1. Clinical and genomic analyses were limited to White Western European ancestry participants due to insufficient sample sizes in other ancestries.

To address drug-specific genetic effects, genetic analyses included overall SSRI responses and drug-specific responses (Citalopram, Fluoxetine, Paroxetine, and Sertraline). All analyses were conducted using R (version 4.2.2). Logistic regressions were performed using the *glm* function (R Core Team, 2021). Associations between PGS and self-reported antidepressant response were reported as the OR for the standardized PGS. Each multivariable model underwent Bonferroni correction; the correction accounted for the total number of predictors (and their levels) assessed in the study (i.e. sociodemographic, clinical, and genetic factors) to allow for comparability across predictor groups (*p* = 0.05/55 = 0.0009). Sensitivity and genetic analyses included an additional correction for multiple testing across six outcomes (*p* = 0.05/330 = 0.0002). Figures display the SSRI response phenotype only. Genetic association plots show SSRI response as well as drug-specific associations.

## Results

### Characteristics

**Figure 1.**
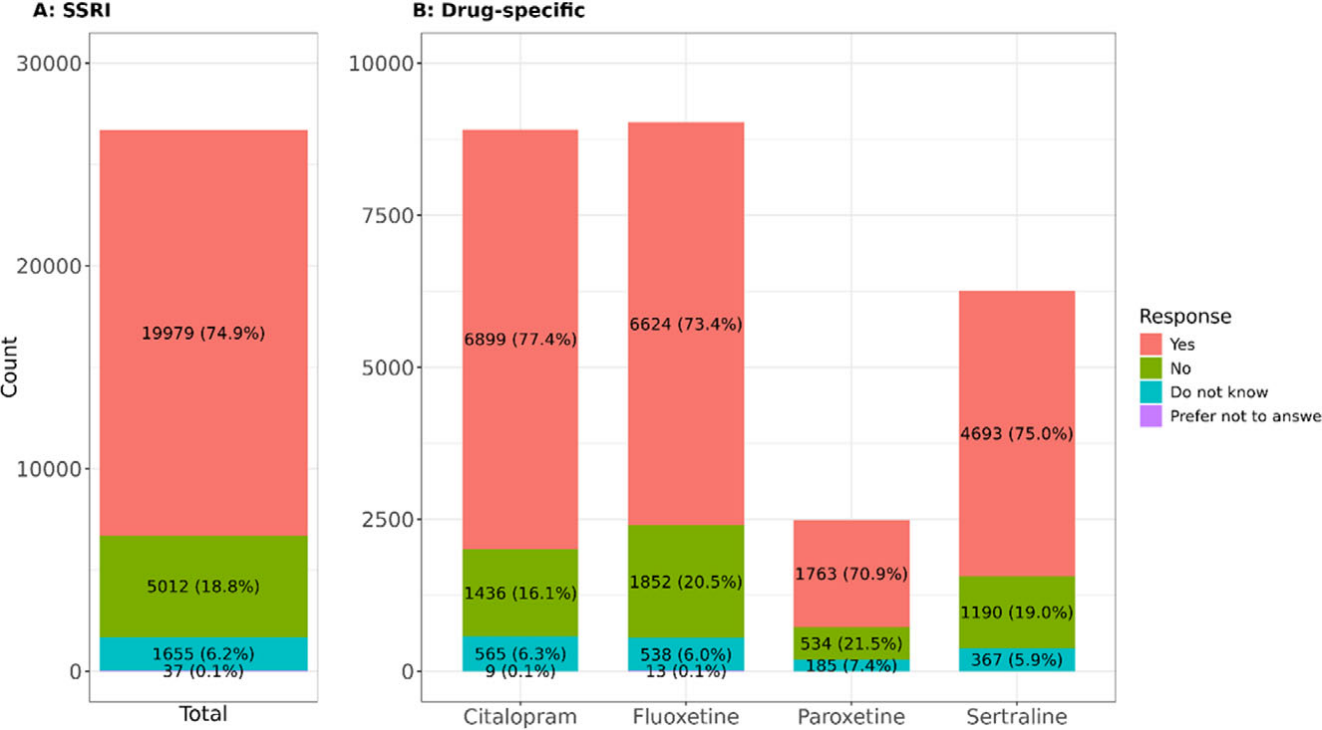
Distribution of the total number of SSRI responses in a subset of UKB participants reporting at least one of the two cardinal symptoms of MDD. SSRI count does not match the number of participants taking SSRIs as some participants reported taking more than one antidepressant. Participants reported ‘Yes’ or ‘No’ that the SSRI drug helped them.

A total of 76,526 individuals with unipolar MDD were included in this analysis. Of these, 45.9% (*N* = 35,088) used prescription medications to alleviate symptoms, and 36.6% (*N* = 27,977) tried antidepressants. About one-quarter (26.9%, *N* = 20,613) used at least one SSRI. Figure 1 presents the total number of SSRI and specific drug counts among SSRI users. Across groups, approximately 7% responded with “Do not know” and <1% with “Prefer not to answer” (Figure 1); these individuals were excluded from further analysis. Final sample sizes were citalopram (*N* = 8335), fluoxetine (*N* = 8476), paroxetine (*N* = 2297), and sertraline (*N* = 5883) (Figure 1). As participants could use multiple SSRIs, the total count for SSRI in Figure 1 exceeds the number of unique participants. To address this, a decision framework (Methods: 2.33) was applied, resulting in 19,516 unique individuals for the SSRI response phenotype and 18,170 individuals for the conservative-SSRI group. Over three-quarters (77.8%) of SSRI individuals tried a single SSRI for at least 2 weeks, with citalopram and fluoxetine being the most common (~35% each), followed by sertraline (21%) and paroxetine (8%). Approximately 5% tried three or more of these SSRIs (Supplementary Data S1, Supplementary Figure S3).

For participants who responded positively to at least one cardinal MDD question, we compared those who had (*N* = 19,516) and had not (*N* = 57,010) tried SSRIs. SSRI users were younger (55.0 ± 7.5 years vs. 52.5 ± 7.3 years, *p* < 1.00 × 10*^−300^*; Table 1), more likely to be female (63.7 vs. 73.6%, *p* = 2.08 × 10^−142^) and of white ethnicity (97.9 vs. 98.5%, *p* = 6.18 × 10^−10^). SSRI users also had lower income (annual household income <£18,000: 13.9 vs. 16.4%, *p* = 1.50 × 10^−23^), came from less deprived neighborhoods (−1.6 vs. −1.4, *p* = 7.98 × 10^−11^) and were less likely to have a university degree (46.3 vs. 43.8%, *p* = 5.17 × 10^−18^).

**Table 1.** Participant characteristics among those who have and have not tried selective serotonin reuptake inhibitors (SSRI) in a subsample of the UKB participants reporting at least one of the two cardinal symptoms for MDD

	SSRI not tried	SSRI tried	
	*N* = 57,010	*N* = 19,516	*p*-value^a^
**Age**			
Mean (SD)	55.0 (7.5)	52.5 (7.3)	<1.00 × 10^−300^
Median	56	52	
Range (min, max)	(40, 72)	(40, 70)	
IQR	(49, 61)	(47, 58)	
**Sex**			
Female (%)	36,287 (64)	14,368 (74)	2.08 × 10^−142^
Male (%)	20,723 (36)	5148 (26)	
**Ethnic background**			
Asian (%)	431 (0.8)	81 (0.4)	6.18 × 10^−10^
Black (%)	375 (0.7)	74 (0.4)	
Mixed (%)	361 (0.6)	130 (0.7)	
White (%)	55,195 (98)	19,081 (99)	
Missing	648	150	
**Annual income**			
<£18,000 (%)	7145 (14)	2947 (16)	1.50 × 10^−23^
£18,000–£31,000 (%)	12,012 (23)	15,038 (23)	
£31,000–£52,000 (%)	15,038 (29)	5399 (30)	
£52,000–£100,000 (%)	13,509 (26)	4407 (25)	
>£100,000 (%)	3780 (7)	1070 (6)	
Missing	5526	1510	
**Educational attainment**			
Secondary (GCSEs, 11 years)	7896 (14)	2959 (15)	5.17 × 10^−18^
Further (A-levels, 13 years)	7838 (14)	2940 (15)	
Vocational (NVQs, BTECs, etc., ≥12 years)	11,142 (20)	3995 (21)	
University (Degree, ≥16 years)	26,275 (46)	8506 (44)	
None listed	3562 (6)	1020 (5)	
Missing	297	96	
**Townsend Deprivation Index (TDI)**			
Mean (SD)	−1.6 (2.9)	−1.4 (3.0)	7.98 × 10^−11^
Median	−2.3	−2.2	
Range (min, max)	(−6.3, 10.5)	(−6.3, 11.0)	
IQR	(−3.75, 0.05)	(−3.67, 0.35)	
Missing	74	29	

^a^ p-values was generated using Pearson’s chi-squared test for categorical variables and the Wilcoxon rank sum test for continuous variables; p < 0.05. GCSE – General Certificate of Secondary Education, A-levels – Advanced Level qualifications, NVQs – National Vocational Qualifications, BTECs – Business and Technology Education Council qualifications.

Drug-specific analysis showed consistent age and sex differences between those who had and had not tried specific SSRIs (e.g. Citalopram: Not tried vs. Tried, Supplementary Data S2, Supplementary Table S6). Participants who tried citalopram, fluoxetine, and sertraline were generally younger than those who had not tried the respective drug (citalopram: 52.9 ± 7.3 vs. 51.9 ± 7.3 years, *p* = 5.1 × 10^−22^; fluoxetine: 52.8 ± 7.3 vs. 52.1 ± 7.1 years, *p* = 3.7 × 10^−12^; sertraline: 52.7 ± 7.1 vs. 51.9 ± 7.5 years, *p* = 9.4 × 10^−16^); while paroxetine users were older (52.4 ± 7.3 vs. 53.2 ± 7.2 years, *p* = 3.4 × 10^−08^).

Further demographic insights revealed fluoxetine users were more likely to be from less deprived neighborhoods and have a university degree (−1.5 ± 3 vs. −1.3 ± 3, *p* = 3.6 × 10^−03^_;_ University degree: 43% vs. 45%, *p* = 5.6 × 10^−04^), while sertraline users were less educated (45% vs. 41%, *p* = 1.6 × 10^−04^), more likely to be non-white (White: 99% vs. 98%, *p* = 0.02), and from lower-income groups (<£18 K: 16% vs. 18%). Paroxetine users had lower incomes (<£18 K: 16% vs. 18%, *p* = 2.2 × 10^−04^) but were more educated (University degree: 43% vs. 51%, *p* = 3.2 × 10^−13^).

### Antidepressant exposure and response

When considering definitive responses to whether drugs had worked for participants (“Y”/“N”), 79.6% felt SSRIs made them “*feel better*” (Supplementary Data S1, Supplementary Table S7). This positive response was consistent across individual drugs, but rates varied by drug (χ^2^ = 73.28, *p* = 8.5 × 10^−16^), highest for citalopram (82.8%) and lowest for paroxetine (76.8%) (Supplementary Data S1, Supplementary Table S8).

### Factors associated with treatment response

#### Sociodemographic factors

In univariable analysis, the strongest factor (largest effect size and lowest *p*-value) associated with SSRI non-response across all phenotypes was alcohol and illicit drug use, followed by male gender, higher neighborhood deprivation, current smoking, lower income (<£18 K), previous drinking, and mixed ethnicity (Supplementary Data S2, Supplementary Table S1).

**Figure 2.**
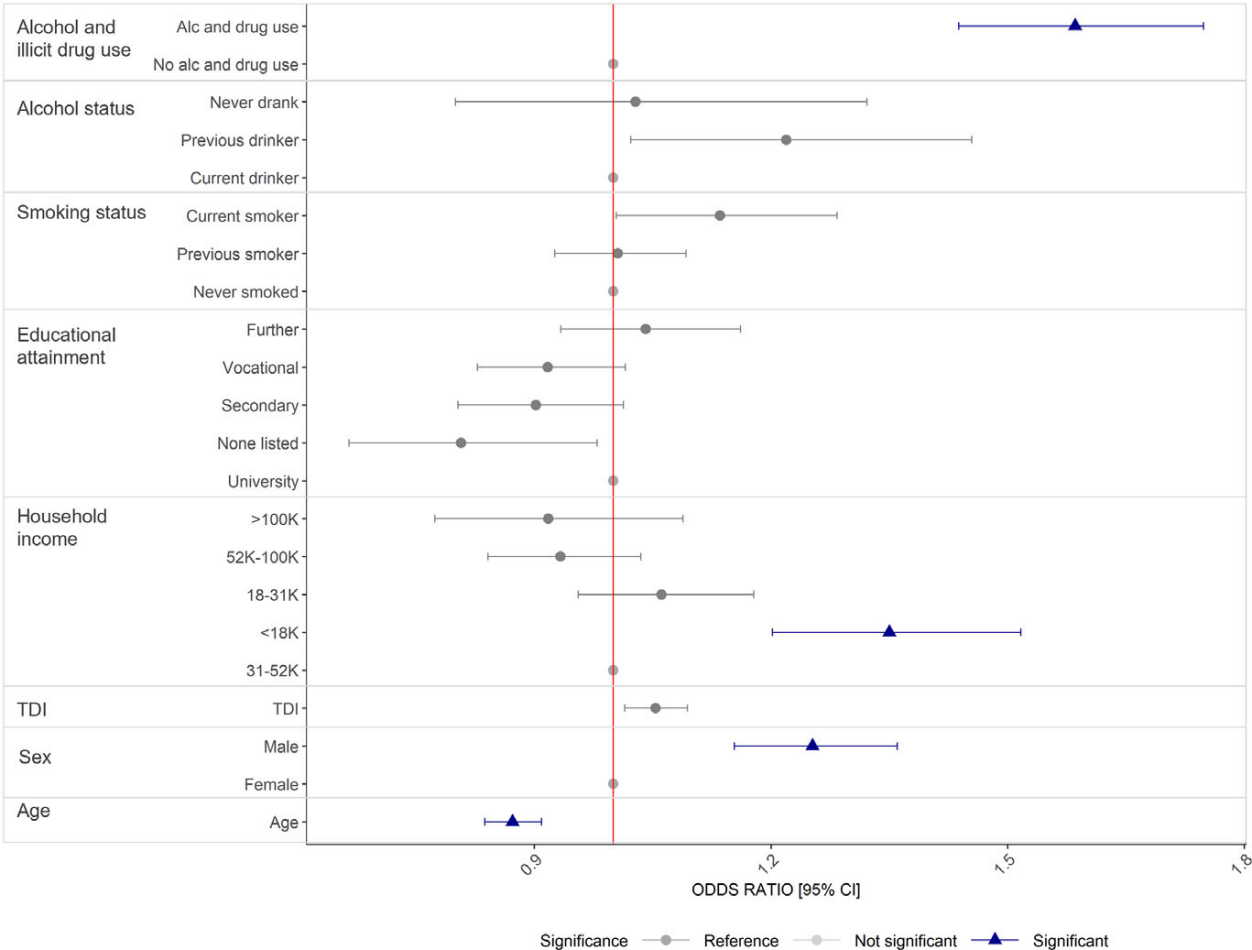
Sociodemographic factors associated with self-reported SSRI non-response in UKB. *N* = 17,479. Odds ratios, 95% confidence intervals significance, controlling for all other factors. Significance based on multiple testing correction (*P* < 0.0009). Ethnic background has been excluded from this figure because of wide confidence intervals (see Supplementary Data S2, Supplementary Table S1).

For the SSRI response outcome, 17,789 participants had complete data across included predictors. Multivariable logistic regression showed alcohol and illicit drug use, age, gender, and income remained significant predictors of SSRI non-response after multiple testing adjustment (Figure 2, Supplementary Data S2, Supplementary Table S2). Specifically, alcohol and illicit drug use was associated with higher odds of reporting that SSRIs did not improve symptoms (OR = 1.59, 95%CI = 1.44–1.75, *p* = 2.23 × 10^−20^) – that is, those who used alcohol and illicit drugs to manage their cardinal MDD symptoms were more likely to report an SSRI did not help them improve (“feel better”) compared to those who did not use alcohol and illicit drugs. Male gender (OR = 1.25, 95%CI = 1.15–1.36, *p* = 8.29 × 10^−08^) and lower income (£18 K) relative to the median income (£30 K–£52 K) (OR = 1.35, 95%CI = 1.20–1.52, *p* = 4.22 × 10^−07^) were also linked to non-response. Compared to older individuals, younger age was associated with a decreased likelihood of SSRI non-response (OR = 0.87, 95%CI = 0.84–0.91, *p* = 8.92 × 10^−11^), with the odds of non-response decreasing by 13% for each additional year.

Neighborhood deprivation, smoking status, drinking status, and educational attainment were nominally significant but did not meet the multiple testing threshold (*p* < 0.0009). These patterns were consistent in the conservative-SSRI outcome (*N* = 16,259) where TDI was also associated with non-response (OR = 1.08, 95%CI = 1.03–1.13, *p* = 8.71 × 10^−04^).

At the drug-specific level (Supplementary Data S1), treatment response associations largely mirrored the broader SSRI pattern, with some variation. Multivariable analysis showed that after correcting for multiple testing (*p* < 0.0002), alcohol and illicit drug use was the strongest predictor of non-response to citalopram (*N* = 7524) (OR = 1.53, 95%CI = 1.31–1.79, *p* = 5.78 × 10^−08^), while male gender was the greatest predictor for fluoxetine (*N* = 7621) (OR = 1.55, 95%CI = 1.37–1.75, *p* = 2.63 × 10^−12^). Younger age was significantly associated with response to citalopram (OR = 0.86, 95%CI = 1.81–0.92, *p* = 9.03 × 10^−05^), and to sertraline (OR = 0.86, 95%CI = 1.80–0.93, *p* = 9.03 × 10^−05^).

#### Clinical factors

In univariable analysis, SSRI non-response was associated with a later age at the first episode, multiple episodes, worst episode duration exceeding 2 years, lack of mood-brightening during the worst episode, difficulty coping with rejection, feelings of heavy limbs, worthlessness, and thoughts of death. Non-response associations were similar and consistent at the SSRI and drug-specific level (Supplementary Data S2, Supplementary Table S3).

**Figure 3.**
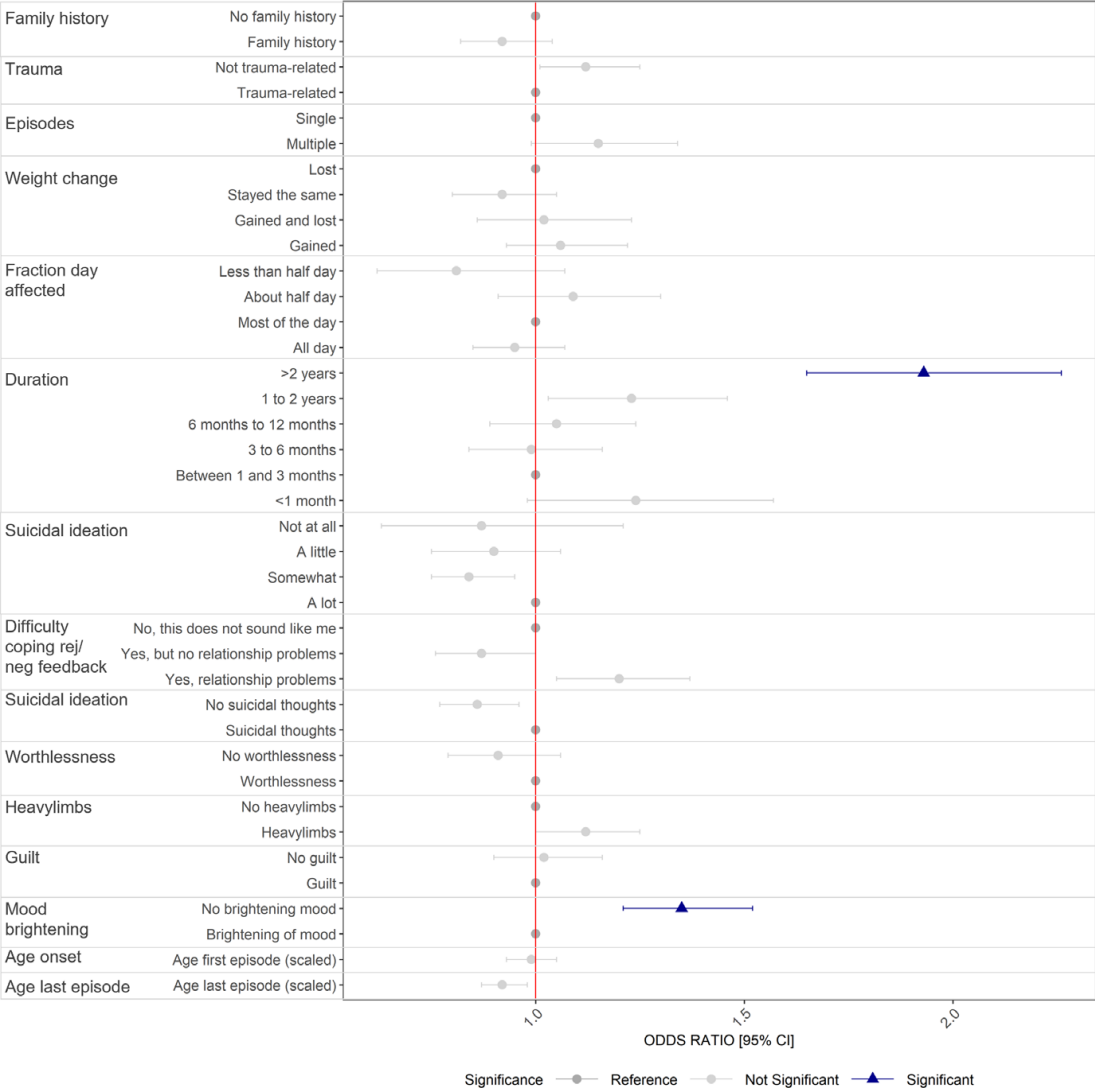
Clinical factors associated with self-reported SSRI non-response in UKB. Odds ratios, 95% confidence intervals significance, controlling for all other factors (*N* = 9418. Analyses have been restricted to those of white ethnic background and adjusted for age and sex. Significance based on multiple testing correction (*P* < 0.0009).

In multivariable analyses (Figure 3, Supplementary Data S2, Supplementary Table S4), SSRI non-response (*N* = 9418) was significantly associated with a worst episode lasting more than 2 years (OR = 1.93, 95%CI = 1.65–2.26, *p* = 3.87 × 10^−16^) and no brightening of mood in response to positive events during the worst episode of depression (OR = 1.35, 95%CI = 1.21–1.52, *p* = 2.37 × 10^−07^). In sensitivity analyses, the worst episode duration over 2 years was associated with non-response for each drug (citalopram: OR = 2.11, 95%CI = 1.64–2.72, *p* = 8.50 × 10^−09^; fluoxetine: OR = 1.65, 95%CI = 1.3–2.1, *p* = 4.29 × 10^−05^; paroxetine: OR = 2.43, 95%CI = 1.55–3.81, *p* = 1.02 × 10^−04^; sertraline: OR = 1.97, 95%CI = 1.49–2.61, *p* = 1.68 × 10^−06^).

#### Genetic factors

##### CYP2C19 metabolizer status

Our study included 18,992 unrelated individuals of European ancestry with available CYP2C19 genotype and phenotype data. The largest group was CYP2C19 normal metabolizers (NM) (7507, 39.5%), followed by rapid metabolizers (RM) (5155, 27.1%) and intermediate metabolizers (IM) (4943, 26.0%). As expected, poor metabolizers (PM) (443, 2.3%) and ultra-rapid (UM) (930, 4.9%) were less common, with 0.1% having indeterminate status. Similar proportions were observed at the drug-specific level (Supplementary Data S1, Supplementary Table S11).

**Figure 4.**
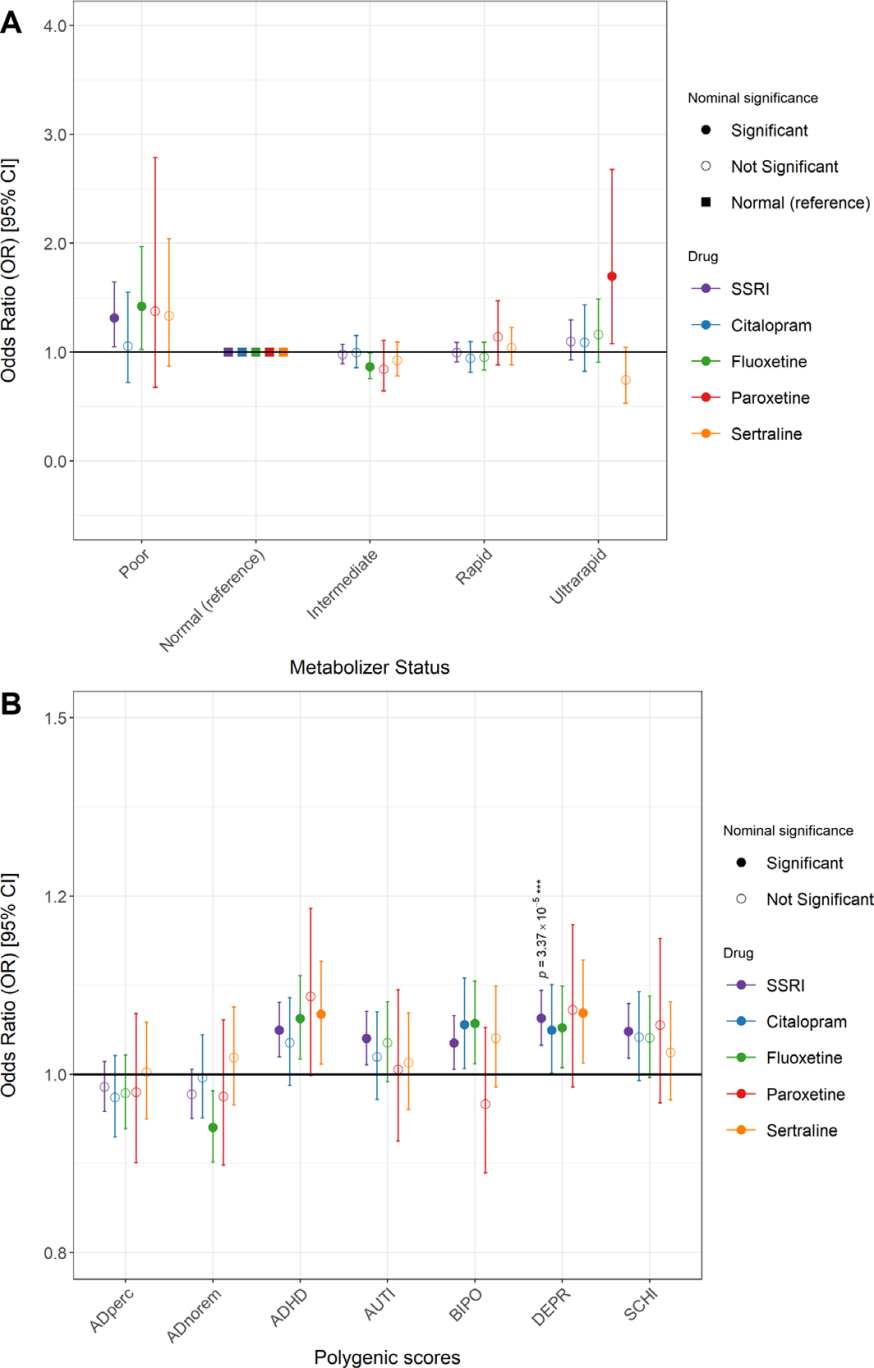
Genetic predictors of self-reported antidepressant non-response in UKB. (A) Inferred CYP2C19 metabolizer status associated with self-reported antidepressant non-response in UKB. Forest plots depict odds ratios and 95% confidence intervals for the association between metabolizer category (compared to normal) and treatment non-response (blue markers) for SSRIs and specific SSRIs (Citalopram, Fluoxetine, Paroxetine, Sertraline). Inferred metabolizer status levels are Poor, Normal (reference), Intermediate, Rapid, and Ultra-rapid. Significance: *p* < 0.05; displayed *p* values indicate significance persisted after multiple testing corrections (*P* < 0.0002). (B) Psychiatric and antidepressant response PGS associated with self-reported antidepressant non-response in UKB. The association analyses between SSRIs and specific SSRIs (Citalopram, Fluoxetine, Paroxetine, Sertraline) and self-reported antidepressant outcomes and various mental health condition and treatment PGS. PGS include DEPR: Depression, ADHD: Attention Deficit Hyperactivity Disorder, AUTI: Autism, BIPO: Bipolar Disorder, SCHI: Schizophrenia. ADperc: Percentage improvement, ADnorem: AD non-remission. Significance: *p* < 0.05; displayed *p* values indicate significance persisted after multiple testing corrections (*P* < 0.0002).

Association analyses between self-reported SSRI response and CYP2C19 metabolizer status showed that those with a PM status had nominally significant higher likelihood of non-response compared to NM (OR = 1.31, 95%CI = 1.05–1.65, *p* = 1.77 × 10^−02^); this was mirrored in the conservative approach but with increased significance (OR = 1.41, 95%CI = 1.09–1.83, *p* = 8.57 × 10^−03^). A similar association was observed for fluoxetine (OR = 1.42, 95%CI = 1.02–1.97, *p* = 3.50 × 10^−02^) (Figure 4A, Supplementary Data S2, Supplementary Table S5). Drug-specific associations showed nominally significant lower odds of self-reported non-response among IM using fluoxetine (OR = 0.87, 95%CI = 0.76 = 0.99, *p* = 3.87 × 10^−02^) and higher odds of non-response among UM taking paroxetine (OR = 1.70, 95%CI = 1.08–2.68, *p* = 2.29 × 10^−02^). No significant associations were noted among citalopram and sertraline users. None of the identified associations survived multiple testing correction.

##### Polygenic scores

We tested for the association between self-reported SSRI response and PGS for five psychiatric conditions and two antidepressant response measures (Figure 4B, Supplementary Data S2, Supplementary Table S6). For psychiatric traits, SSRI (“SSRI” in Figure 4) non-response was nominally associated with all psychiatric condition PGS: depression (OR = 1.08, 95%CI = 1.04–1.12, *p* = 3.37 × 10^−05^), attention deficit hyperactivity disorder (ADHD) (OR = 1.06, 95%CI = 1.02–1.10, *p* = 1.06 × 10^−03^), autism (OR = 1.05, 95%CI = 1.01–1.09, *p* = 7.42 × 10^−03^), bipolar (OR = 1.04, 95%CI = 1.01–1.08, *p* = 2.06 × 10^−02^), and schizophrenia (OR = 1.06, 95%CI = 1.02–1.10, *p* = 1.53 × 10^−03^). In each case, higher genetic liability to the condition (higher PGS) corresponded with a lack of response to SSRIs (6–8% increase in the odds of non-response per SD increase of PGS). The phenotypic variance explained (*R2*) for each of these PGS with SSRI non-response ranged between 0.07% and 0.16% (Supplementary Data S1, Supplementary Figure S4). Only the association with the PGS for depression survived multiple testing corrections. For the antidepressant response PGS (non-remission (ADnorem) and percentage improvement (ADperc), no significant findings were noted.

Sensitivity analyses similarly showed the conservative approach similarly revealed all disorder PGS to be nominally associated with non-response but only the PGS for ADHD survived multiple testing correction. In the drug-specific non-response analysis, other disorders showed nominal significance (e.g. depression and bipolar PGS with citalopram and fluoxetine; and depression and ADHD PGS with sertraline) (Figure 4B). For the antidepressant response PGS (non-remission [ADnorem] and percentage improvement [ADperc]), a nominally significant finding was noted between fluoxetine non-response and the non-remission PGS (*R*^2^ = 0.235, OR = 0.93, 95%CI = 0.88–0.98, *p* = 4.80 × 10^−03^). Across all drugs, none of these findings persisted following multiple testing correction.

## Discussion

Antidepressants, particularly SSRIs, are widely prescribed, yet remission rates remain low, and few risk factors guide personalized prescribing. A major challenge in identifying factors to predict treatment is the absence of response measures that are scalable across large sample sizes. Several large studies have applied patient self-report questionnaires on response, asking a single, simple question about whether an antidepressant has ‘helped’. In this article, we assessed the UKB’s antidepressant self-report data to identify clinical, sociodemographic, and genetic factors associated with a lack of response to SSRIs, aiming to demonstrate that this self-reported outcome is a valid measure of antidepressant response.

In UKB, 75% of participants reported a positive SSRI response, substantially higher than the 35% remission rate noted in trials (Rush et al., 2004). This discrepancy may reflect the question wording (“Has <drugname> helped you feel better” with possible responses of “Yes, at least a little,” and “No”), which is not synonymous with remission. This is a lower threshold than the criteria used for remission in clinical trials, where depression symptoms must fall below the diagnostic threshold for depression (Stone et al., 2022). Here, the antidepressant may be providing symptom reduction rather than remission and may also coincide with the natural progression of an episode. The perception of antidepressant efficacy based on improved daily functioning aligns with findings that patients prioritize a return to normal functioning as much as the absence of symptoms (Zimmerman et al., 2006). Additionally, unlike the 8–12-week assessment span of clinical trials, the UKB questionnaire uses a retrospective measure based on the full length of an SSRI treatment period, suggesting while self-reported response may not adequately gauge ‘remission’ status, it could reflect improvements in personal functioning.

For the self-reported antidepressant response to be a valuable phenotype, a deeper understanding of factors associated with a negative response is needed. Our analysis revealed significant associations with sociodemographic, clinical, and genetic variables, with consistent direction and effect sizes across SSRIs. Non-response to SSRIs was associated with being male, older age, having a lower income and alcohol and illicit drug use. These factors largely align with clinical trial predictors while also highlighting real-world influences like substance use.

For example, males exhibited lower response rates, consistent with some clinical studies (Gibiino, Marsano, & Serretti, 2014; Serretti, Gibiino, & Drago, 2011; Trivedi et al., 2006), although no difference was found in iSPOT-D (Saveanu et al., 2015) or a meta-analysis of Randomized Control Trials (RCTs) (Cuijpers et al., 2014). Lower-income has been associated with reduced treatment response in a recent RCT (Mills et al., 2022), and in STAR*D, where reduced adherence and shorter treatment duration were found in lower-income groups, even when controlled for treatment access and level of care (Jakubovski & Bloch, 2014). Younger age at UKB recruitment was associated with better SSRI response; similar to other research (Uher et al., 2009). This also aligns with data suggesting depression is more chronic and antidepressants less effective in older patients (Haigh, Bogucki, Sigmon, & Blazer, 2018; Strawn et al., 2023). However, no information on the age at antidepressant use is available in UKB.

Clinical associations of poor SSRI response in UKB were persistent low mood and long depressive episodes (≥2 years), consistent with findings that greater severity (Rush et al., 2004) and melancholic depression hinder recovery and reduce perceived antidepressant efficacy (Valerio, Szmulewicz, & Martino, 2018). Hieronymus et al. emphasize targeting low mood to improve MDD treatment outcomes; their SSRI RCT reanalysis found higher rates of efficacy when focusing on mood improvements (where 91% of participants showed efficacy) than with HDRS-17 scores (efficacy in 44%) (Hieronymus, Emilsson, Nilsson, & Eriksson, 2016). Higher severity of depression, as assessed by the proxy measures of a long-duration episode (≥2 years) and by a higher genetic liability for MDD, was correlated with non-response. In clinical trials, more severe depression has a better response (compared to placebo), but the different measurements of response and severity make a direct comparison of these results difficult.

Our study provides insights into the genetic basis for SSRI response. Higher genetic liability for depression, and attention-deficit/hyperactivity disorder (ADHD) was associated with poorer self-reported antidepressant response, consistent with previous research (Pain et al., 2022). The association between higher PGS and treatment non-response is consistent with clinical studies that link higher depression PGS to increased side effects, such as dizziness and reduced sexual desire, which may impact treatment effectiveness by hindering effective dosage maintenance (Campos et al., 2021). The link between genetic liability for ADHD in UKB may reflect missed diagnosis in this older age group. A recent study reported that 28% of adults referred for mood and anxiety assessments had undiagnosed ADHD, with the number of prior SSRI prescriptions being a significant predictor (Sternat, Mohammed, & Furtado, 2016).

CYP2C19 metabolizer status proportions in our study were highly concordant with other studies in European populations (Campos et al., 2022; Ionova et al., 2020). A nominally significant positive association between poor metabolizer (PM) status and SSRI non-response was observed. This association was mirrored in the conservative approach and amongst fluoxetine users, but no association survived multiple testing corrections. This finding contrasts with previous studies reporting increased efficacy of SSRIs, such as citalopram and sertraline, among PMs (Fabbri et al., 2018; Li et al., 2024). Authors suggest that the broad therapeutic windows of these medications may limit the influence of CYP2C19 polymorphisms on treatment response (Campos et al., 2022). Also, CYP2C19 is not the sole enzyme involved in SSRI metabolism: while it plays a primary role in metabolizing citalopram, its role is less significant for sertraline, fluoxetine, and paroxetine (Li et al., 2024; Obach, Cox, & Tremaine, 2005; Yuce-Artun et al., 2016). The non-response for PMs may also reflect high adverse events in this group, where participants stopped taking the drug due to severe side effects (and so report ‘no’ that the drug did not help them). In contrast, in clinical trials, only PMs reaching the end of the trial period are included, which may exclude those with severe adverse events.

At the drug-specific level, the nominal significance of PM status among fluoxetine’s users may be in part due to fluoxetine’s relatively long half-life, which averages 2–4 days for the parent compound and extends to 7–15 days for its active metabolite, norfluoxetine (Hiemke & Härtter, 2000). In contrast, citalopram has a much shorter half-life of approximately 36 h (Hiemke & Härtter, 2000). The significantly longer half-life of fluoxetine can lead to substantial drug accumulation, particularly in poor metabolizers, potentially increasing the likelihood of side effects and influencing treatment adherence and response. Non-response to paroxetine in UM suggests insufficient drug levels to achieve therapeutic effects, necessitating higher doses (Li et al., 2024; Wong et al., 2023).

Mental health research increasingly recognizes the importance of patient perspectives and priorities, but there is a paucity of studies on patients’ views on antidepressant use and response. The Wellcome Trust has prioritized patient and public involvement (PPI) in all their funded Mental Health studies (Wellcome Trust, n.d.), and our Antidepressant Medications: Biology, Exposure, and Response (AMBER) research program has ongoing research to understand the priorities of those taking antidepressants.

Self-report questionnaires from the UKB provide novel and rich data to better understand patient perspectives, which can, in turn, help more accurately measure outcomes and tailor management pathways. Despite its potential utility, additional measures, such as a refined scale that captures multiple types of positive responses, could further enhance our understanding of what constitutes meaningful recovery for patients.

This study has many strength in its patient-centered approach, providing valuable information on antidepressant treatment from the patient’s perspective. This easily collected phenotype is available in large numbers in the UKB, the Australian Genetics of Depression Study (AGDS; Byrne et al., 2020), and Genetic Links to Anxiety and Depression (GLAD; Davies et al., 2019; Koch et al., 2024). Our study shows good alignment of sociodemographic and clinical risk factors between the UKB self-report response and clinical trial research in antidepressants, suggesting both measures pick up common information on response and non-response. This provides an important source of information to expand sample sizes for research into the genetic underpinnings of antidepressant response. Differences between clinician-rated and self-rated measures suggest patients have unique perspectives on treatment outcomes, which are critical to a complete assessment of depression (Campos et al., 2022; Ramanuj, Ferenchick, & Pincus, 2019; Uher et al., 2012; Zimmerman et al., 2006).

While our study offers valuable insights into antidepressant response, it has several limitations. The analyses were based on UKB, which has better-than-average socioeconomic circumstances than the general population, potentially limiting the generalizability of the findings and underestimating the impact of low income. The reliance on retrospective self-reports is subject to recall bias and inaccuracies – participants may either underreport or overreport their past symptoms and healthcare experiences. Sociodemographic variables were reported at the time of the baseline UKB questionnaire, not during the participant’s worst depressive episode, complicating factor assessment. Further limitations of this analysis include its focus on those who have tried SSRIs, potentially resulting in a dataset that may not fully capture the diversity of antidepressant usage amongst the UK population. Moreover, the inability to determine the timing of antidepressant exposure and whether the drugs were used independently or concurrently, or whether they were prescribed for different episodes or had overlapping use, complicates the interpretation of treatment patterns and outcomes. We are unable to dissect the natural disorder course from the antidepressant response, and some patients responding that the drug helped them may have had a similar recovery trajectory without an antidepressant.

It is important to note that many individuals reporting improvement in response to antidepressants may be reacting to nonspecific aspects of treatment rather than the pharmacological effects of antidepressants, as reflected in high placebo response rates in trials. While metabolizer status is likely to reflect drug-specific effects, other predictors (e.g. low income) may identify individuals at greater risk of poor outcomes irrespective of the specific treatment received. This distinction between predictors of response and true moderators highlights a key limitation and underscores the need for further research in this area.

## Conclusion

Self-reported antidepressant response outcome in UK Biobank is influenced by sociodemographic, clinical characteristics, and common genetic variation, similar to clinical response measures. Despite the higher frequency of positive response outcomes compared to clinical trials, these retrospective self-report outcomes replicate known associations with current antidepressant treatment outcomes. This suggests that self-reported outcomes, while measuring a positive response that is not equivalent to remission, capture meaningful aspects of antidepressant effectiveness, particularly from the patient’s perspective.

## Supporting information

Kamp et al. supplementary material 1 Kamp et al. supplementary material

Kamp et al. supplementary material 2 Kamp et al. supplementary material

## Data Availability

Code relating to the analyses in this article is available on GitHub (https://github.com/michellekamp/Predictors-of-Self-reported-SSRI-response) and the GenoPred Pipeline code is available from the GenoPred GitHub Code Repository (https://opain.github.io/GenoPred/). Summary statistics from the PGC are available online (https://pgc.unc.edu/for-researchers/download-results/). Data from the UK Biobank (https://www.ukbiobank.ac.uk) are available to bona fide researchers upon application.

## Supplementary material

The supplementary material for this articlecan be found at http://doi.org/10.1017/S0033291725000388.
