# Supplementary material for: Sociodemographic, clinical, and genetic factors associated with self-reported antidepressant response outcomes in the UK Biobank
Source: Psychol Med. 2025 Mar 12;55:e80. doi: 10.1017/S0033291725000388 (PMC12080641; doi:10.1017/S0033291725000388)
Supplement: Kamp et al. supplementary material 1 — Kamp et al. supplementary material [file S0033291725000388sup001.docx]

**Supplementary Materials 1**

**Sociodemographic, clinical, and genetic factors associated with self-reported antidepressant response outcomes in the UK Biobank.**

Michelle Kamp, Chris Wai Hang Lo, Grigorios Kokkinidis, Mimansa Chauhan, Alexandra C. Gillett, AMBER Research Team, Andrew M. McIntosh, Oliver Pain, and Cathryn M. Lewis

**Contents**

[**Phenotype identification** 2](#_Toc187655495)

[**Table S1: MHQ2 questionnaire UKB data fields used to identify SSRI users** 2](#_Toc187655496)

[**Study Participants** 3](#_Toc187655497)

[**Figure S1: Flowchart of UK Biobank participants included in antidepressant outcome analyses** 3](#_Toc187655498)

[**UK Biobank genotype data** 4](#_Toc187655499)

[**Table S2: Decision framework for defining antidepressant response outcomes among participants using at least one Selective Serotonin Reuptake Inhibitor (SSRI)** 6](#_Toc187655500)

[**Table S3: Count and proportions off individuals within each antidepressant response outcome group among participants using at least one Selective Serotonin Reuptake Inhibitor (SSRI)** 6](#_Toc187655501)

[**Clinical symptoms and characteristics** 7](#_Toc187655502)

[**Table S4: Depression clinical characteristics and associated UK Biobank data fields from the MHQ2 questionnaire** 7](#_Toc187655503)

[**GWAS summary statistics of Polygenic score analysis** 9](#_Toc187655504)

[**Table S5: GWAS summary statistics used to develop five psychiatric disorder-related PGS scores and two antidepressant response-associated trait scores** 9](#_Toc187655505)

[**Association of assessment centre** 10](#_Toc187655506)

[**Table S6: Association test results between self-reported antidepressant response and UKB Assessment centre** 10](#_Toc187655507)

[**Multivariable model Variance Inflation Factors (VIF) analysis** 11](#_Toc187655508)

[**Table S7: Variance inflation Factors (VIF) in regression analyses assessing self-reported antidepressant response with sociodemographic factors** 11](#_Toc187655509)

[**Table S8: Variance inflation Factors (VIF) in regression analyses assessing self-reported antidepressant response with clinical factors** 12](#_Toc187655510)

[**Antidepressant response and exposure** 13](#_Toc187655511)

[**Figure S2: Upset plot showing number of participants trying different SSRI combinations in the UK Biobank.** 13](#_Toc187655512)

[**Table S11: Sample distribution of inferred metabolizer status across selective serotonin reuptake inhibitors (SSRI).** 14](#_Toc187655513)

[**Phenotypic variance explained of self-reported antidepressant non-response by psychiatric and antidepressant response PGS in UK Biobank** 15](#_Toc187655514)

# **Phenotype identification**

## **Table S1: MHQ2 questionnaire UKB data fields used to identify SSRI users**

| **UKB field ID** | **Item** | **Answers** |
| --- | --- | --- |
| 29011 | Have you ever had a time in your life when you felt sad, blue, or depressed for two weeks or more in a row? | Yes  No  Do not know  Prefer not to answer |
| 29012 | Have you ever had a time in your life lasting two weeks or more when you lost interest in most things like hobbies, work, or activities that usually give you pleasure? | Yes  No  Do not know  Prefer not to answer |
| 29038 | Have you ever tried the following for these problems? | Unprescribed medication (more than once)  Medication prescribed to participant (for at least two weeks)  Drugs or alcohol (more than once)  None of the options listed  Prefer not to answer |
| 29039 | Have you ever tried any of the following medications for at least two weeks? | Citalopram (sometimes called Cipramil)  Fluoxetine (Prozac or Oxactin)  Sertraline (Lustral)  Paroxetine (Seroxat)  Amitriptyline (Elavil)  Dosulepin (Prothiaden)  Other antidepressant(s)  Do not know  Prefer not to answer |
| 29040 | Has citalopram helped you to feel better? | Yes, at least a little  No  Do not know  Prefer not to answer |
| 29041 | Has fluoxetine helped you to feel better? |  |
| 29042 | Has sertraline helped you to feel better? |  |
| 29043 | Has paroxetine helped you to feel better? |  |

**UKB – UK Biobank*

# **Study Participants**

**
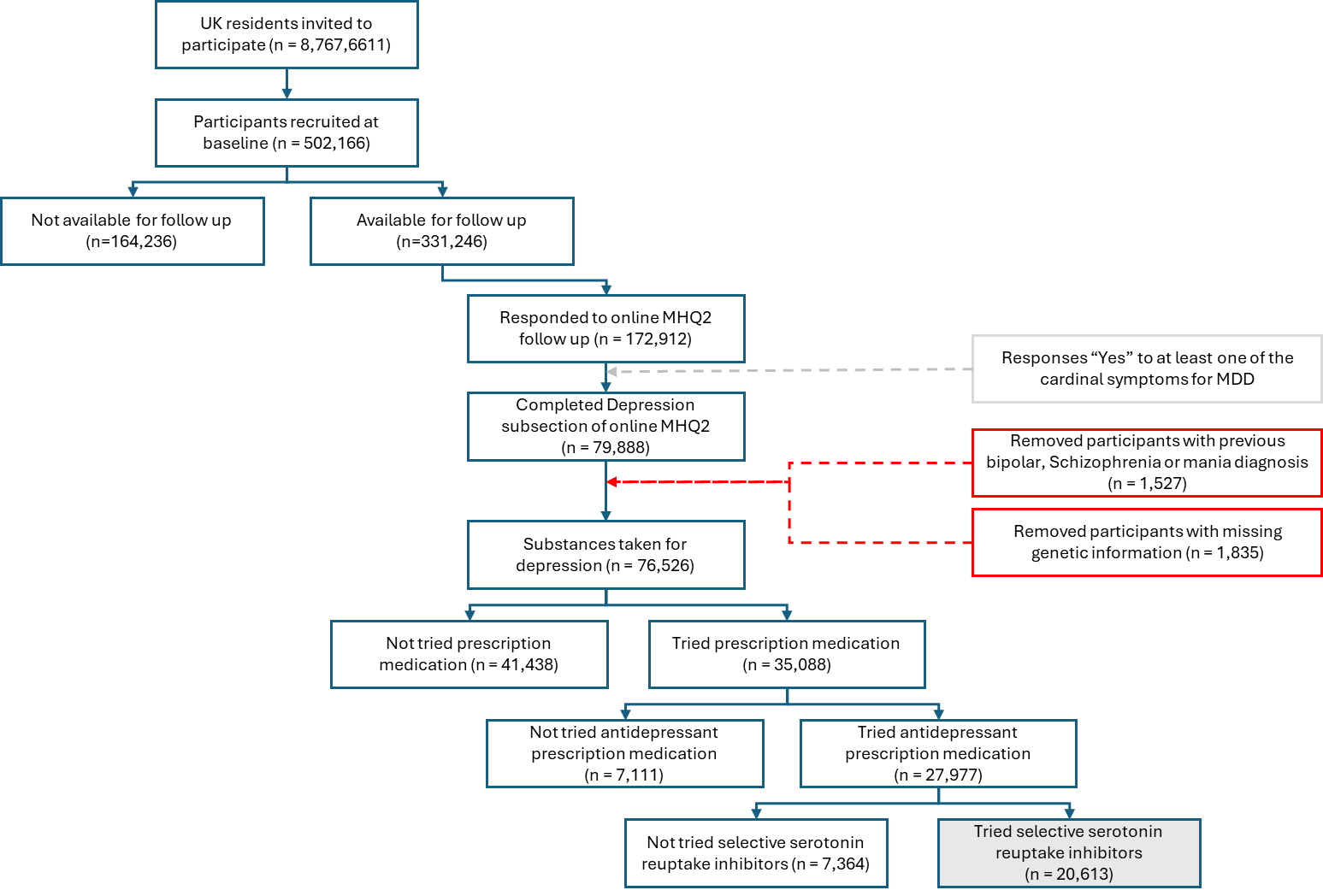
**

**Figure S1****: Flowchart of UK Biobank participants included in antidepressant outcome analyses**

## **UK Biobank genotype data**

In the UK Biobank (UKB), 488,377 participants were genotyped using either the UKB Axiom or UK BiLEVE Axiom arrays, covering 805,426 single nucleotide polymorphisms (SNPs) (Bycroft et al., 2018). SNPs underwent a rigorous quality control process, which included removing SNPs with more than 5% missing calls, a minor allele frequency (MAF) below 0.0001, and those that failed quality checks in multiple batches. After these initial quality assessments, 670,739 SNPs were successfully imputed for 487,442 participants using SHAPEIT3 (Bycroft et al., 2018). This imputation corrected for missing genotypes and sample heterozygosity issues. Haplotype imputation utilized both the Haplotype Reference Consortium (HRC) (McCarthy et al., 2016) and a merged reference panel from UK10K and the 1000 Genomes Project Phase 3 (Huang et al., 2015). This comprehensive approach yielded a final count of 93,095,623 autosomal SNPs that met the quality standards for the study.

Additional quality controls were applied directly to the genotyped data from the UKB. SNPs with a genotyping missing rate greater than 2%, a Hardy-Weinberg Equilibrium (HWE) p-value less than 10^-8^, and a MAF less than 1% were excluded. Participants were also screened for anomalies in heterozygosity that were identified during centralized quality control processes. Population structure was evaluated using four-means clustering on the first two principal components, retaining only individuals of European descent. The relatedness among individuals was managed in further analyses using REGENIE, which helped refine the study's robustness (Mbatchou et al., 2021). The imputed SNPs went through an additional filtration step, requiring an imputation quality (INFO) score of at least 0.4 to be considered reliable for subsequent analyses.

**References**

Bycroft, C., Freeman, C., Petkova, D., Band, G., Elliott, L. T., Sharp, K., … Young, A. (2018). The UK Biobank resource with deep phenotyping and genomic data. Nature, 562, 203–209.

Huang, J., Howie, B., McCarthy, S., Memari, Y., Walter, K., Min, J. L., … Consortium, U. (2015). Improved imputation of low-frequency and rare variants using the UK10K haplotype reference panel. Nature Communications, 6(1), 8111. doi: 10.1038/ncomms9111

Mbatchou, J., Barnard, L., Backman, J., Marcketta, A., Kosmicki, J. A., Ziyatdinov, A., … Marchini, J. (2021). Computationally efficient whole-genome regression for quantitative and binary traits. Nature Genetics, 53(7), 1097–1103. doi: 10.1038/s41588-021-00870-7

McCarthy, S., Das, S., Kretzschmar, W., Delaneau, O., Wood, A. R., Teumer, A., … Durbin, R. (2016). A reference panel of 64,976 haplotypes for genotype imputation. Nature Genetics, 48(10), 1279–1283. doi: 10.1038/ng.3643

**Decision framework: self-reported SSRI response phenotypes**

This decision framework (detailed in Table S2) accounted for exposure to multiple drugs and aggregated responses across the four SSRIs. Participants' responses were categorised based on their exposure to one, two, three, or four SSRIs:

- Single SSRI Exposure: Participants reporting a positive response ("Y") were classified as responders, while those reporting no response ("N") were classified as non-responders.
- Two SSRI Exposures: Participants with two positive responses ("YY") were classified as responders, and those with two negative responses ("NN") were classified as non-responders. Mixed responses ("YN") were classified as non-responders as most people respond “Y,” and “N” are minority, or marked as missing data (NA) if classification was uncertain.
- Three SSRI Exposures: Participants with three positive responses ("YYY") were classified as responders, and those with three negative responses ("NNN") were classified as non-responders. Mixed responses (e.g., "YNN", "YYN") were generally classified as non-responders, particularly if the majority response was negative, or marked as missing data (NA) if uncertain.
- Four SSRI Exposures: Participants with four positive responses ("YYYY") were classified as responders, and those with four negative responses ("NNNN") were classified as non-responders. Mixed responses (e.g., "YNNN", "YYNN", "YYYN") were generally classified as non-responders, particularly if the majority response was negative, or marked as missing data (NA) if uncertain.

## **Table S2: Decision framework for defining antidepressant response outcomes among participants using at least one Selective Serotonin Reuptake Inhibitor (SSRI)**

| **Number of drugs** | **Drug specific outcomes** | **SSRI phenotype** | **SSRI conservative**  **phenotype** |
| --- | --- | --- | --- |
| 1 | Y | Y | Y |
|  | N | N | N |
| 2 | YY | Y | Y |
|  | YN | N | NA |
|  | NN | N | N |
| 3 | YYY | Y | Y |
|  | YYN | N | NA |
|  | YNN | N | N |
|  | NNN | N | N |
| 4 | YYYY | Y | Y |
|  | YYYN | Y | NA |
|  | YYNN | N | N |
|  | YNNN | N | N |
|  | NNNN | N | N |

## **Table S3:** **Count and proportions off individuals within each antidepressant response outcome group among participants using at least one Selective Serotonin Reuptake Inhibitor (SSRI)**

| **Number of drugs** | **Drug specific outcomes** | **SSRI phenotype** | **Proportion of sample** |
| --- | --- | --- | --- |
| 1 | Y | Y | 15 191 (77.8%) |
|  | N | N |  |
| 2 | YY | Y | 3 329 (17.1%) |
|  | YN | N |  |
|  | NN | N |  |
| 3 | YYY | Y | 842 (4.3%) |
|  | YYN | N |  |
|  | YNN | N |  |
|  | NNN | N |  |
| 4 | YYYY | Y | 154 (0.8%) |
|  | YYYN | Y |  |
|  | YYNN | N |  |
|  | YNNN | N |  |
|  | NNNN | N |  |

# **Clinical symptoms and characteristics**

## **Table S4: Depression clinical characteristics and associated UK Biobank data fields from the MHQ2 questionnaire**

| **MDD characteristics** | **Field ID** | **Response options^a^** | **Reference category** |
| --- | --- | --- | --- |
| Depression possibly related to stressful or traumatic event | 29013 | - No - Yes | Yes |
| Fraction of day affected during worst episode | 29014 | - All day long - Most of the day - About half of the day - Less than half of the day | Most of the day |
| Frequency of depressed days during worst episode | 29015 | - Every day - Almost every day - Less often | Almost every day |
| Brightening of mood in response to positive events during worst episode | 29016 | - No - Yes | Yes |
| Time of day that mood was worse during worst episode. | 29017 | - In the morning - In the evening or at night - Mood did not vary | Mood did not vary |
| Feelings of tiredness during worst episode of depression | 29018 | - No - Yes | Yes |
| Feelings of heaviness in limbs during worst episode of depression | 29019 | - No - Yes | No |
| Change in appetite during worst episode of depression | 29020 | - No change in appetite - Increased appetite - Decreased appetite | Decreased appetite |
| Weight change during worst episode of depression | 29021 | - Gained weight - Lost weight - Both gained and lost some weight during this time - Stayed about the same or was on a diet | Lost weight |
| Difficulty concentrating during worst episode of depression | 29026 | - No - Yes | Yes |
| Feelings of worthlessness during worst period of depression | 29027 | - No - Yes | Yes |
| Feelings of guilt during worst period of depression | 29028 | - No - Yes | Yes |
| Thoughts of death during worst depression episode | 29029 | - No - Yes | Yes |
| Duration of worst depression | 29030 | - Less than a month - Between 1 and 3 months - Over 3 months, but less than 6 months - Over 6 months, but less than 12 months - 1 to 2 years - Over 2 years | Between 1 and 3 months |
| Impact on normal roles during worst period of depression | 29031 | - A lot - Somewhat - A little - Not at all | A lot |
| Difficulty coping with rejection or negative responses | 29032 | - Yes, caused problems in work/social relationships - Yes, but no problems in work/social relationships - No, this does not sound like me | No, this does not sound like me |
| Lifetime number of depressed periods | 29033 | Continuous number scale binarized to:   - Single - Multiple ( >1 episode) | Single |
| Age at first episode of depression | 29034 | Continuous number scale | NA |
| Depression possibly related to childbirth | 29035 | - No - Yes |  |
| Age at last episode of depression | 29036 | Continuous number scale | NA |
| Has/did your father ever suffer from? | 20107 | - Hip fracture - Prostate cancer - Severe depression - Parkinson's disease - Alzheimer's disease/dementia - Diabetes - High blood pressure - Chronic bronchitis/emphysema - Breast cancer - Bowel cancer - Lung cancer - Stroke - Heart disease | NA |
| Has/did your mother ever suffer from? | 20110 |  |  |
| Has/did your father ever suffer from? | 20111 |  |  |

^a^ All data field IDs have “Prefer not to answer” and “I do not know” as a response option

# **GWAS summary statistics of Polygenic score analysis**

## **Table S5: GWAS summary statistics used to develop five psychiatric disorder-related PGS scores and two antidepressant response-associated trait scores**

| **Phenotype** | **Abbr.** | **PMID** | **Authors (Ref)** | **N** | **Case_N** | **Control_N** |
| --- | --- | --- | --- | --- | --- | --- |
| Major Depressive disorder | DEPR | 29700475  (excl. UKB and 23andMe) | Wray et al., 2018 (Wray et al., 2018) | 143,265 | 45,591 | 97,674 |
| Attention Deficit/ Hyperactivity disorder | ADHD | 30478444 | Demontis et al., 2019 (Demontis et al., 2019) | 55,374 | 20,183 | 35,191 |
| Autism | AUTI | 30804558 | Grove et al., 2019 (Grove et al., 2019) | 48,350 | 18,381 | 29,969 |
| Bipolar | BIPO | 31043756 | Stahl et al., 2019 (Stahl et al., 2019) | 147,172 | 9,412 | 137,760 |
| Schizophrenia | SCHI | 29483656 | Pardiñas et al., 2018 (Pardiñas et al., 2018) | 35,802 | 11,260 | 24,542 |
| Antidepressant-non remission | AD_non-rem_ | 35712048 | Pain et al., 2022 (Pain et al., 2022) | 5151 | 3,299 | 1,852 |
| Antidepressant-percentage improvement | AD_perc_ | 35712048 | Pain et al., 2022 (Pain et al., 2022) | 5218 | 5218 | 0 |

# **Association of assessment centre**

## **Table S6: Association test results between self-reported antidepressant response and UKB Assessment centre**

| **Assessment centre** | **SSRI**  **N = 19516** | | **SSRI cons**  **N = 18170** | | **Citalopram**  **N = 8335** | | **Fluoxetine**  **N = 8476** | | **Paroxetine**  **N = 2297** | | **Sertraline**  **N = 5883** | |
| --- | --- | --- | --- | --- | --- | --- | --- | --- | --- | --- | --- | --- |
|  | **OR [95% CI]** | ***p*** | **OR [95% CI]** | ***p*** | **OR [95% CI]** | ***p*** | **OR [95% CI]** | ***p*** | **OR [95% CI]** | ***p*** | **OR [95% CI]** | ***p*** |
| Barts | 1.19 [0.95-1.51] | 0.137 | 1.22[0.93-1.60] | 0.155 | 1.23[0.84-1.80] | 0.282 | 1.02[0.72-1.45] | 0.911 | 0.63[0.32-1.24] | 0.182 | 1.28[0.82-1.98] | 0.281 |
| Birmingham | 1.11[0.92-1.35] | 0.267 | 1.18[0.94-1.47] | 0.149 | 1.06[0.78-1.45] | 0.721 | 1.08[0.80-1.46] | 0.618 | 0.82[0.48-1.41] | 0.471 | 1.05[0.75-1.47] | 0.764 |
| Bristol | 0.98[0.83-1.15] | 0.778 | 1.02[0.84-1.24] | 0.849 | 0.97[0.74-1.27] | 0.802 | 0.99[0.77-1.26] | 0.928 | 1.02[0.63-1.64] | 0.946 | 1.04[0.77-1.40] | 0.815 |
| Bury | 0.94[0.77-1.14] | 0.513 | 0.83[0.65-1.07] | 0.148 | 0.75[0.54-1.05] | 0.098 | 0.99[0.74-1.34] | 0.958 | 0.95[0.53-1.68] | 0.847 | 1.02[0.71-1.46] | 0.912 |
| Cardiff | 1.17[0.95-1.44] | 0.148 | 1.14[0.89-1.46] | 0.312 | 1.06[0.76-1.49] | 0.720 | 1.24[0.91-1.70] | 0.175 | 1.39[0.79-2.44] | 0.257 | 1.29[0.87-1.90] | 0.202 |
| Croydon | 1.08[0.89-1.30] | 0.459 | 1.12[0.90-1.41] | 0.301 | 1.19[0.87-1.63] | 0.264 | 1.05[0.79-1.38] | 0.752 | 0.92[0.53-1.60] | 0.772 | 0.81[0.56-1.18] | 0.277 |
| Edinburgh | 1.01[1.80-1.26] | 0.948 | 0.82[0.61-1.08] | 0.161 | 1.31[0.89-1.92] | 0.171 | 0.79[0.58-1.07] | 0.129 | 0.94[0.51-1.64] | 0.834 | 1.38[0.88-2.15] | 0.158 |
| Glasgow | 1.15[0.93-1.43] | 0.197 | 1.06[0.82-1.38] | 0.660 | 1.17[0.82-1.68] | 0.378 | 1.16[0.86-1.57] | 0.331 | 0.73[0.38-1.40] | 0.348 | 1.00[0.65-1.52] | 0.993 |
| Hounslow | 1.07[1.89-1.30] | 0.464 | 1.08[0.86-1.35] | 0.496 | 0.98[0.71-1.35] | 0.896 | 1.11[0.84-1.46] | 0.467 | 1.14[0.69-1.88] | 0.615 | 1.08[0.76-1.54] | 0.672 |
| Liverpool | 1.04[0.87-1.24] | 0.675 | 1.09[0.89-1.35] | 0.401 | 1.16[0.86-1.56] | 0.322 | 0.92[0.70-1.20] | 0.544 | 0.92[0.56-1.53] | 0.759 | 1.06[0.78-1.46] | 0.696 |
| Manchester | 1.18[0.94-1.48] | 0.144 | 1.08[0.82-1.42] | 0.586 | 1.04[0.71-1.53] | 0.849 | 1.18[0.85-1.63] | 0.328 | 0.71[0.37-1.36] | 0.303 | 1.12[0.75-1.68] | 0.573 |
| Middlesborough | 1.02[0.83-1.25] | 0.853 | 0.97[0.76-1.23] | 0.775 | 0.82[0.57-1.18] | 0.284 | 0.99[0.75-1.32] | 0.967 | 0.86[0.46-1.59] | 0.627 | 1.13[0.80-1.58] | 0.482 |
| Newcastle | 1.07[0.90-1.27] | 0.473 | 1.04[0.84-1.27] | 0.725 | 1.16[0.87-1.56] | 0.315 | 0.90[0.70-1.15] | 0.408 | 1.14[0.69-1.88] | 0.601 | 1.06[0.78-1.45] | 0.710 |
| Nottingham | 0.93[0.77-1.11] | 0.409 | 0.95[0.77-1.18] | 0.653 | 0.93[0.69-1.26] | 0.646 | 0.96[0.74-1.24] | 0.737 | 0.98[0.58-1.67] | 0.946 | 0.92[0.66-1.27] | 0.601 |
| Oxford | 1.13[0.90-1.42] | 0.304 | 1.19[0.71-1.09] | 0.210 | 1.36[0.94-1.95] | 0.099 | 0.88[0.63-1.25] | 0.484 | 0.56[0.26-1.19] | 0.132 | 1.08[0.70-1.67] | 0.720 |
| Reading | 0.96[0.80-1.16] | 0.702 | 0.95[0.824.09] | 0.655 | 1.07[0.79-1.44] | 0.668 | 0.89[0.67-1.16] | 0.383 | 0.67[0.38-1.19] | 0.173 | 1.04[0.74-1.46] | 0.813 |
| Sheffield | 0.97[0.81-1.15] | 0.703 | 0.88[0.64-1.14] | 0.237 | 0.94[0.69-1.29] | 0.703 | 0.74[0.58-0.96] | 0.023 | 1.10[0.67-1.82] | 0.696 | 1.10[0.80-0.50] | 0.568 |
| Stockport | 1.70[0.83-3.47] | 0.146 | 1.83[0.82-4.09] | 0.141 | 1.66[0.59-4.65] | 0.337 | 1.56[0.54-4.56] | 0.413 | 1.54[0.14-17.37] | 0.729 | 1.77[0.45-6.96] | 0.412 |
| Stoke | 0.98[0.78-1.24] | 0.858 | 0.85[0.61-1.14] | 0.280 | 0.89[0.61-1.30] | 0.540 | 0.77[0.54-1.11] | 0.160 | 0.92[0.51-1.68] | 0.789 | 1.02[0.67-1.56] | 0.930 |
| Swansea | 0.93[0.53-1.62] | 0.801 | 0.60[0.27-1.33] | 0.211 | 1.42[0.68-2.95] | 0.346 | 0.57[1.20-1.67] | 0.309 | 1.45E-06[0-4.27E244] | 0.964 | 0.77[0.29-2.03] | 0.592 |
| Wrexham | 0.55[0.19-1.59] | 0.271 | 0.41[0.10-1.73] | 0.224 | 0.36[0.05-2.72] | 0.319 | 1.53[0.47-5.03] | 0.484 | 1.45E-06[0-inf] | 0.988 | 0.32[0.04-2.45] | 0.272 |
| Leeds (ref) | 1.0[1.0-1.0] | NA | 1.0[1.0-1.0] | NA | 1.0[1.0-1.0] | NA | 1.0[1.0-1.0] | NA | 1.0[1.0-1.0] | NA | 1.0[1.0-1.0] | NA |

# **Multivariable model Variance Inflation Factors (VIF) analysis**

## **Table S7: Variance inflation Factors (VIF) in regression analyses assessing self-reported antidepressant response with sociodemographic factors**

|  |  |
| --- | --- |
| \| **Group** \| **Predictor** \| **Cit** \| **Flu** \| **Par** \| **Ser** \| **SSRI** \| **SSRI_cons** \| \| --- \| --- \| --- \| --- \| --- \| --- \| --- \| --- \| \| All \| Sex \| 1.03 \| 1.03 \| 1.05 \| 1.03 \| 1.03 \| 1.03 \| \|  \| Age (scaled) \| 1.11 \| 1.12 \| 1.14 \| 1.16 \| 1.13 \| 1.14 \| \|  \| Ethnic background \| 1.03 \| 1.03 \| 1.03 \| 1.04 \| 1.03 \| 1.03 \| \|  \| Annual income \| 1.29 \| 1.25 \| 1.23 \| 1.29 \| 1.27 \| 1.28 \| \|  \| Highest education \| 1.17 \| 1.15 \| 1.17 \| 1.17 \| 1.16 \| 1.16 \| \|  \| TDI (scaled) \| 1.14 \| 1.13 \| 1.13 \| 1.17 \| 1.13 \| 1.13 \| \|  \| Alcohol status \| 1.05 \| 1.04 \| 1.05 \| 1.04 \| 1.04 \| 1.05 \| \|  \| Smoking status \| 1.12 \| 1.13 \| 1.11 \| 1.15 \| 1.12 \| 1.11 \| \|  \| Alcohol and illicit drug use \| 1.07 \| 1.08 \| 1.09 \| 1.08 \| 1.07 \| 1.07 \| |  |
|  |  |

*Cit – Citalopram, Flu – Fluoxetine, Par – Paroxetine, Ser – Sertraline, SSRI – Composite-SSRI, SSRI-conservative response phenotype*

## **Table S8: Variance inflation Factors (VIF) in regression analyses assessing self-reported antidepressant response with clinical factors**

| **Predictor** | **Cit 1** | **Cit 2** | **Flu 1** | **Flu 2** | **Par 1** | **Par 2** | **Ser 1** | **Ser 2** | **SSRI 1** | **SSRI 2** | **SSRI cons** | **SSRI_cons** |
| --- | --- | --- | --- | --- | --- | --- | --- | --- | --- | --- | --- | --- |
| Age first episode | 1.36 | 1.35 | 1.33 | 1.33 | 1.49 | 1.48 | 1.36 | 1.36 | 1.34 | 1.35 | 1.38 | 1.38 |
| Age last episode | 1.14 | 1.14 | 1.14 | 1.13 | 1.13 | 1.13 | 1.11 | 1.11 | 1.13 | 1.13 | 1.13 | 1.13 |
| Brightening of mood | 1.38 | 1.33 | 1.38 | 1.33 | 1.34 | 1.28 | 1.37 | 1.31 | 1.36 | 1.31 | 1.37 | 1.31 |
| Change in appetite | 3.16 | - | 3.46 | - | 4.08 | - | 2.92 |  | 3.14 |  | 3.06 | - |
| Difficulty coping rejection | 1.29 | 1.27 | 1.28 | 1.28 | 1.29 | 1.28 | 1.30 | 1.28 | 1.27 | 1.26 | 1.28 | 1.27 |
| Feelings of guilt | 1.39 | 1.39 | 1.38 | 1.39 | 1.43 | 1.41 | 1.44 | 1.43 | 1.40 | 1.40 | 1.42 | 1.42 |
| Feeling of heavy limbs | 1.12 | 1.11 | 1.12 | 1.11 | 1.12 | 1.12 | 1.11 | 1.11 | 1.11 | 1.10 | 1.12 | 1.11 |
| Feeling worthlessness | 1.47 | 1.46 | 1.44 | 1.44 | 1.52 | 1.54 | 1.39 | 1.40 | 1.45 | 1.45 | 1.47 | 1.48 |
| Roles impacted | 1.40 | 1.35 | 1.34 | 1.31 | 1.48 | 1.41 | 1.39 | 1.34 | 1.35 | 1.31 | 1.37 | 1.33 |
| Duration of worst episode | 1.22 | 1.19 | 1.24 | 1.22 | 1.33 | 1.28 | 1.22 | 1.19 | 1.19 | 1.17 | 1.20 | 1.18 |
| Fraction of day affected | 1.93 | 1.46 | 1.80 | 1.43 | 1.81 | 1.45 | 2.10 | 1.45 | 1.91 | 1.43 | 1.92 | 1.44 |
| Frequency of depressed days | 1.86 | - | 1.68 | - | 1.76 | - | 1.97 | - | 1.82 | - | 1.83 | - |
| Thoughts of death | 1.14 | 1.15 | 1.15 | 1.15 | 1.21 | 1.20 | 1.15 | 1.14 | 1.14 | 1.14 | 1.15 | 1.15 |
| Weight change | 3.22 | 1.20 | 3.51 | 1.22 | 4.06 | 1.27 | 2.95 | 1.23 | 3.17 | 1.19 | 3.10 | 1.20 |
| Episodes | 1.35 | 1.35 | 1.36 | 1.36 | 1.43 | 1.40 | 1.31 | 1.31 | 1.35 | 1.35 | 1.40 | 1.40 |
| Depression related to trauma | 1.11 | 1.10 | 1.10 | 1.10 | 1.16 | 1.15 | 1.10 | 1.09 | 1.11 | 1.10 | 1.11 | 1.11 |
| Family history | 1.03 | 1.03 | 1.03 | 1.02 | 1.06 | 1.05 | 1.03 | 1.03 | 1.02 | 1.02 | 1.02 | 1.02 |
| Age | 1.15 | 1.14 | 1.13 | 1.12 | 1.21 | 1.19 | 1.17 | 1.16 | 1.13 | 1.12 | 1.13 | 1.12 |
| Sex | 1.08 | 1.07 | 1.10 | 1.09 | 1.15 | 1.12 | 1.10 | 1.08 | 1.09 | 1.07 | 1.09 | 1.08 |

*Cit – Citalopram, Flu – Fluoxetine, Par – Paroxetine, Ser – Sertraline, SSRI – Composite-SSRI, SSRI-conservative response phenotype*

*Columns show VIF values before (e.g. Cit 1) and after (Cit 2) removing correlated variables, removed variable indicated by '-'.

# **Antidepressant response and exposure**

**
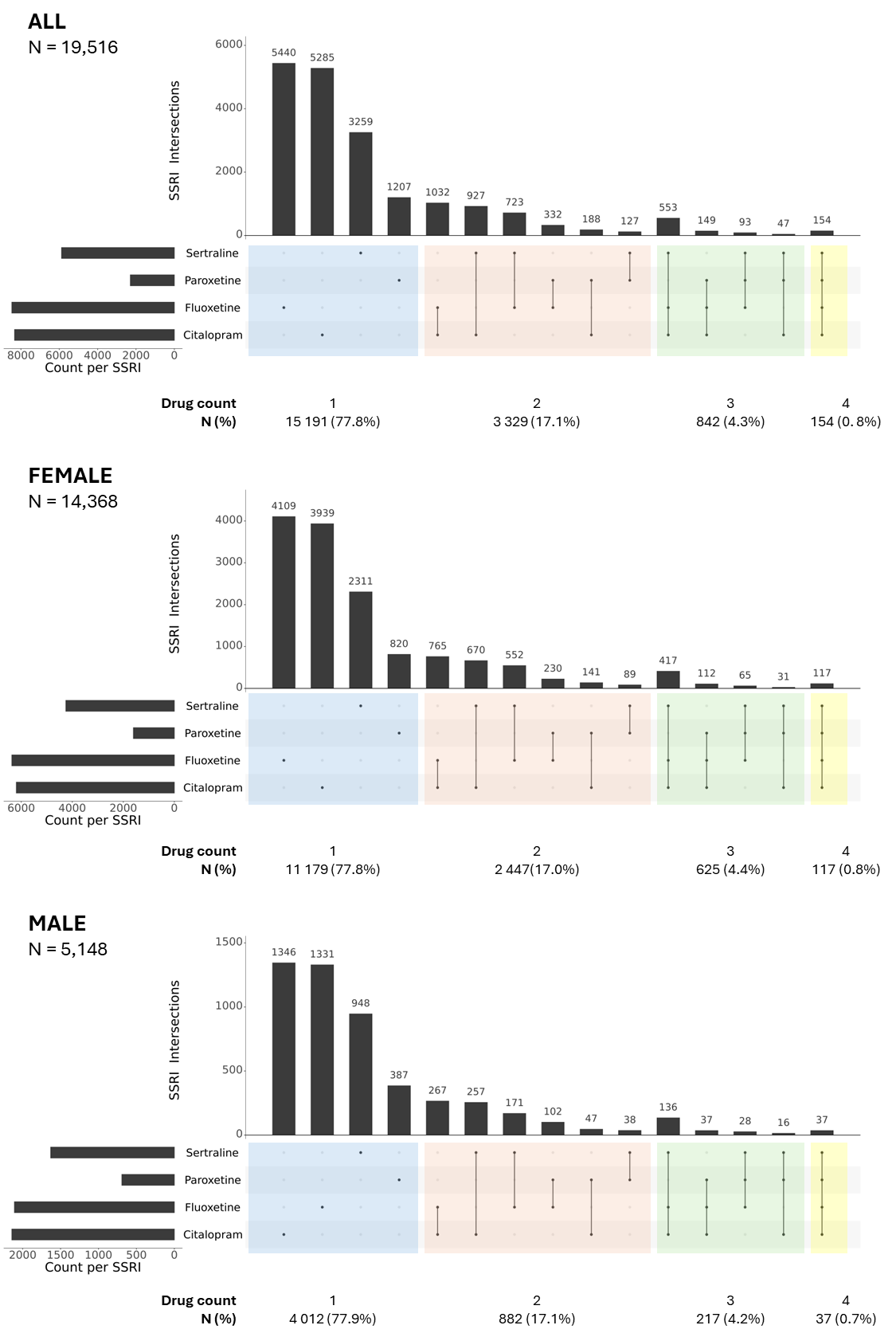
**

## **Figure S2: Upset plot showing number of participants trying different SSRI combinations in the UK Biobank.**

Counts derived from those who responded “Yes”, “No”, to whether the drug (Citalopram, Fluoxetine, Paroxetine and Sertraline) had made them feel better. *SSRI – Selective serotonin reuptake inhibitors.*

**Table S9: Self-reported SSRI response rate comparison across drugs**

| **Group** | **Drug** | **Yes (%)** | **No (%)** |
| --- | --- | --- | --- |
| All | SSRI | 79. 6 | 20.4 |
| Females | SSRI | 80.7 | 19.3 |
| Males | SSRI | 76.5 | 20.4 |

**Table S10: Drug-specific response proportions and chi-squared test results**

| **Group** | **Drug** | **Yes (%)** | **No (%)** | **χ2 stat** | **Df** | **Std res: No** | **Std res: Yes** | ***P*** |
| --- | --- | --- | --- | --- | --- | --- | --- | --- |
| All | Citalopram | 82.8 | 17.2 | 73.28 | 3 | -7.89 | 7.89 | 8.483e-16 |
|  | Fluoxetine | 78.2 | 21.8 |  |  | 5.08 | -5.08 |  |
|  | Paroxetine | 76.8 | 23.2 |  |  | 4.01 | -4.01 |  |
|  | Sertraline | 79.8 | 20.2 |  |  | 0.38 | -0.38 |  |

## **Table S11: Sample distribution of inferred metabolizer status across selective serotonin reuptake inhibitors (SSRI).**

| **Metaboliser status** | **SSRI**  N (%) | **SSRI-cons**^a^  N (%) | **Citalopram**^b^  N (%) | **Fluoxetine**^b^  N (%) | **Paroxetine**^b^  N (%) | **Sertraline**^b^  N (%) |
| --- | --- | --- | --- | --- | --- | --- |
| Poor | 443 (2.3) | 411 (2.3) | 197 (2.4) | 197 (2.4) | 47 (2.1) | 128 (2.2) |
| Normal | 7 507 (39.5) | 6 984 (39.5) | 3 225 (39.7) | 3 258 (39.5) | 853 (382) | 2 270 (39.7) |
| Intermediate | 4 943 (26.0) | 4 596 (26.0) | 2 143 (26.4) | 2 144 (26.0) | 584 (26.1) | 1 480 (25.9) |
| Rapid | 5 155 (27.1) | 4 806 (27.2) | 2 162 (26.6) | 2 247 (27.3) | 637 (28.5) | 1 536 (26.9) |
| Ultra rapid | 930 (4.9) | 862 (4.9) | 391 (4.8) | 393 (4.8) | 111 (5.0) | 293 (5.1) |
| Indeterminate | 14 (0.1) | 13 (0.1) | 9 (0.1) | 5 (0.1) | 2 (0.1) | 4 (0.1) |
| Total N | 18 992 | 17 672 | 8 127 | 8 224 | 2 234 | 5 711 |

^a^ SSRI-cons – SSRI-conservative response phenotype. ^b^ Sample sizes across SSRIs do not add to the total SSRI sample size as some participants reported taking more than one antidepressant.

# **Phenotypic variance explained of self-reported antidepressant non-response by psychiatric and antidepressant response PGS in UK Biobank**


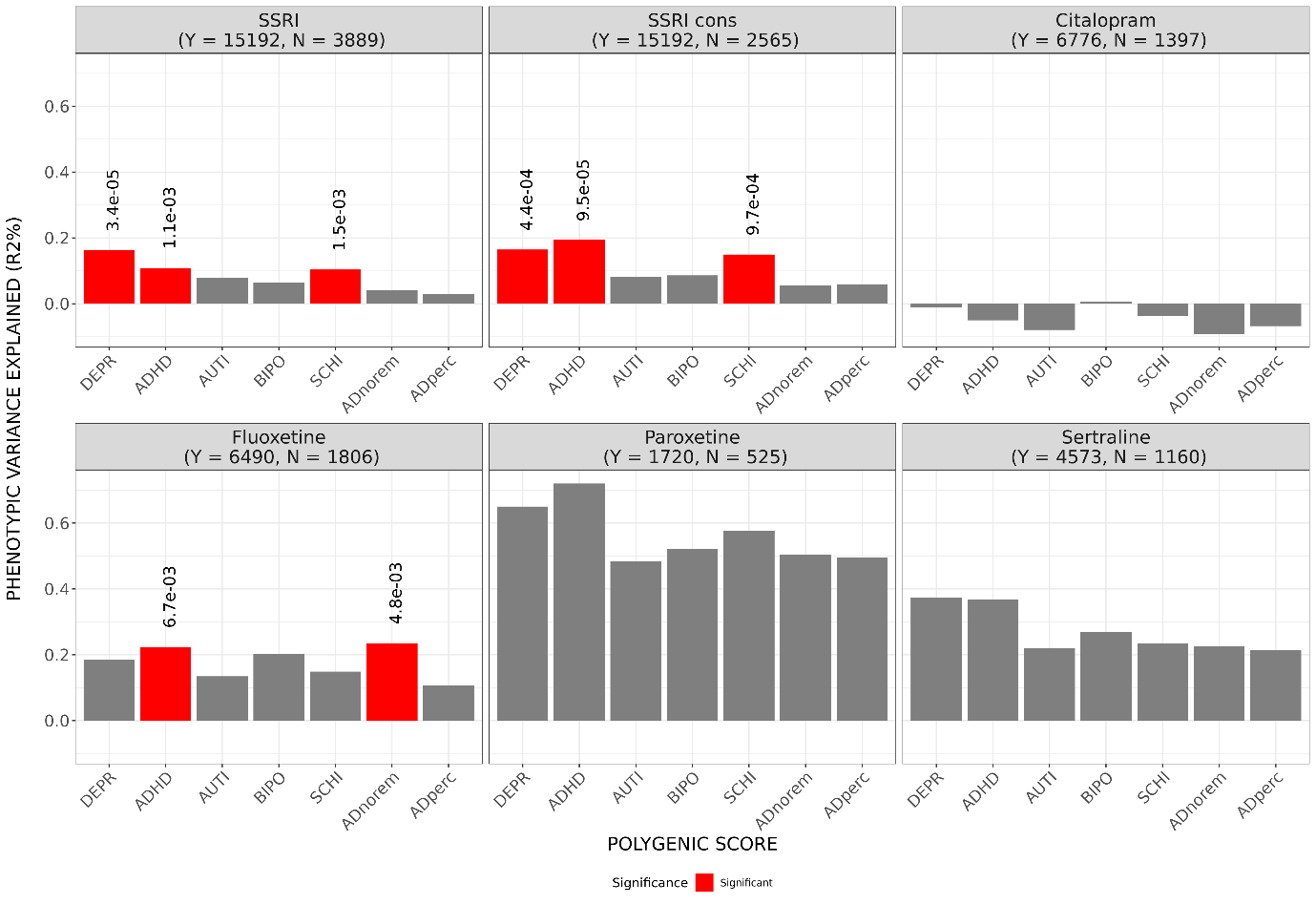


**Figure S3: Phenotypic variance explained (R2%) of self-reported antidepressant non-response by psychiatric and antidepressant response PGS in UK Biobank.** Variance of self-reported antidepressant non-response explained by various mental health condition and treatment polygenic scores between (PGS) for different antidepressants: SSRIs and specific SSRIs (Citalopram, Fluoxetine, Paroxetine, Sertraline). PGS include DEPR: Depression, ADHD: Attention Deficit Hyperactivity Disorder, AUTI: Autism, BIPO: Bipolar Disorder, SCHI: Schizophrenia. ADperc: Percentage improvement, ADNorem: AD non-remission. Multiple testing correction across PGS, within each SSRI P < 0.007.
